# Novel Bisquaternary Oximes—Reactivation of Acetylcholinesterase and Butyrylcholinesterase Inhibited by Paraoxon

**DOI:** 10.3390/molecules14124915

**Published:** 2009-12-01

**Authors:** Kamil Kuca, Lucie Musilova, Jiri Palecek, Vladimir Cirkva, Martin Paar, Kamil Musilek, Martina Hrabinova, Miroslav Pohanka, Jana Zdarova Karasova, Daniel Jun

**Affiliations:** 1 Faculty of Military Health Sciences, University of Defence, Hradec Kralove, Czech Republic; 2 Faculty of Pharmacy in Hradec Kralove, Charles University in Prague, Hradec Kralove, Czech Republic; 3 Institut für Organische Chemie and Zentrum für Biomolekulare Wirkstoffe (BMWZ), Leibniz Universität Hannover, Hannover, Germany; 4 Institute of Chemical Process Fundamentals of the ASCR, v. v. i., Prague, Czech Republic; 5 Faculty of Environmental Sciences, Czech University of Life Sciences Prague, Praha, Czech Republic

**Keywords:** acetylcholinesterase, butyrylcholinesterase, reactivator, nerve agent, oxime, pesticide, scavenger

## Abstract

Four novel bisquaternary aldoxime cholinesterase reactivators differing in their chemical structure were prepared. Afterwards, their biological activity was evaluated for their ability to reactivate acetylcholinesterase (AChE; EC 3.1.1.7) and butyryl-cholinesterase (BuChE; EC 3.1.1.8) inhibited by paraoxon. Their reactivation activity was compared with standard reactivators—pralidoxime, obidoxime and HI-6—which are clinically used at present. As it resulted, none of the prepared compounds surpassed obidoxime, which is considered to be the most potent compound if used for reactivation of AChE inhibited by paraoxon. In case of BuChE reactivation, two compounds (K053 and K068) achieved similar results as obidoxime.

## Introduction

Acetylcholinesterase (AChE; EC 3.1.1.7) reactivators are a group of drugs originally developed as antidotes for the treatment of nerve agent poisonings [1]. They are administered by soldiers using autoinjectors in case of need as immediate help if they are intoxicated by nerve agents [2]. With the increasing demands on the agricultural production, several kinds of pesticides are being extensively used. Among them, organophosphorus pesticides play a very important role [3]. Unfortunately, these compounds act biochemically very similarly to nerve agents (e.g., sarin). They inhibit the enzymes AChE and butyrylcholinesterase (BuChE; 3.1.1.8) [2,4]. If AChE is considered, its inhibition is a life threatening process, because AChE terminates nerve impulses on the synaptic clefts of the nerve system [2]. After the inhibition, it cannot degrade the neuromediator acetylcholine (ACh), ACh cumulates on the synaptic clefts, it overstimulates receptors and the intoxicated organism can die because of cholinergic crisis [2].

**Figure 1:**
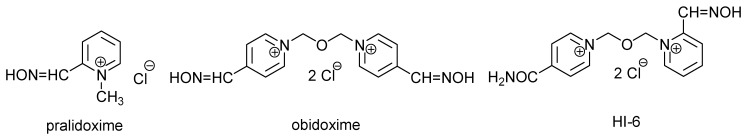
Chemical structures of clinically used acetylcholinesterase reactivators.

Standard therapy of such intoxications consists of administration of anticholinergic drugs (mostly atropine), AChE reactivators (pralidoxime, obidoxime, HI-6 are clinically used; Figure 1) and anticonvulsives (diazepam or avizafone) [5]. The choice of anticholinergics and anticonvulsives is relatively resistant to changes. On the contrary, many new derivatives among the group of AChE reactivators are described. 

At the end of the 20th century, novel approaches for the pre-treatment of nerve agent intoxications, bioscavengers, were investigated. Bioscavengers (cholinesterases, phosphotriesterase, or human paraoxonase) could neutralize nerve agents in the blood stream before they reach their physiological targets. The most investigated, human serum BuChE (EC 3.1.1.8), can be used successfully with relatively high protective potency [6,7].

**Figure 2:**
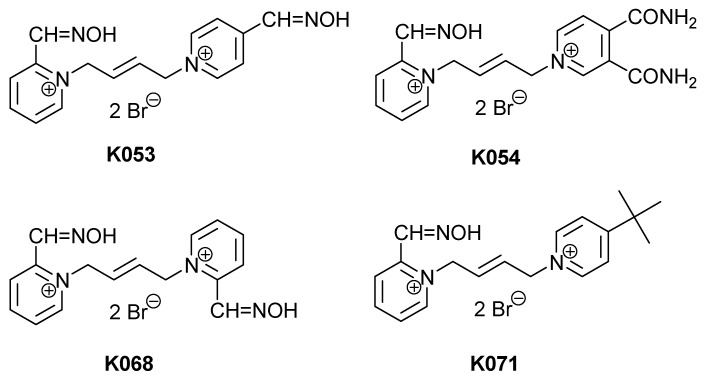
Chemical structures of novel acetylcholinesterase reactivators.

In this study, we have prepared four novel bisquaternary aldoxime cholinesterase reactivators (K053, K054, K068 and K071) with the aim of obtaining new more promising oxime candidates which could in future replace the clinically used reactivators (Figure 2). Paraoxon (POX) was selected as an appropriate organophosphate inhibitor of cholinesterases in our experiments. The newly prepared compounds were also tested for their reactivation of BuChE. BuChE reactivation is at present time well-investigated to get a so-called “pseudocatalytic scavenger” able to act as prophylaxis or treatment of nerve agent poisonings [8,9,10].

## Results and Discussion

**Figure 3:**
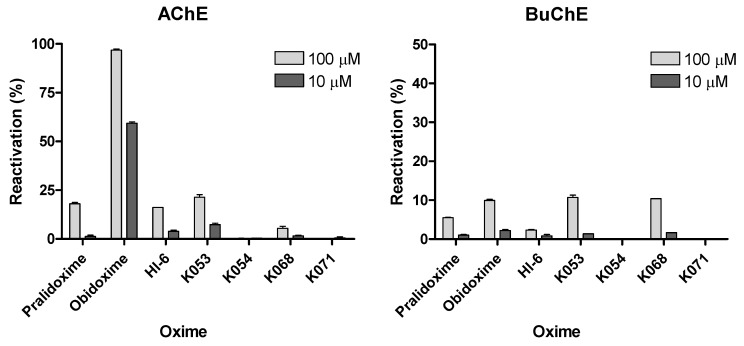
Reactivation of paraoxon-inhibited AChE and BuChE by the novel bisquaternary aldoxime reactivators.

All obtained results are summarized in Table 1 and for better visualization also in Figure 3. As resulted, obidoxime was the most potent reactivator in treatment of paraoxon-inhibited AChE at both concentrations tested (10 µM and 100 µM). Newly prepared oxime K053 together with pralidoxime and HI-6 reached comparable results which are considered to be satisfactory for survival of the intoxicated organism [2]. All other evaluated oximes were not effective in case of AChE reactivation. In case of BuChE reactivation, much more bad results were obtained. This result corresponds with the general finding described already earlier, that reactivation of BuChE is very difficult and different to that for AChE [11,12]. In this case, obidoxime and two novel oximes (K053 and K068) achieved reactivation around 10%. No other oximes (including pralidoxime and HI-6) were able to reactivate sufficiently the POX-inhibited BuChE.

If the obtained results are compared, the new AChE reactivators are not better than the clinically used ones, so that their further investigation cannot be recommended. Due to this, novel structurally different oximes derived from clinically used ones (especially from obidoxime) should be designed and tested for their reactivation potency against POX. On the contrary, if BuChE reactivation is considered, only two oximes (K053 and K068) achieved 10% reactivation. Due to this, if novel BuChE reactivators are to be designed in the future, the results of this study could be used as first approximation to the desired structure with higher BuChE reactivation potency.

## Experimental

### General

All chemicals used in this study were of reagent grade. They were obtained from commercial sources (Sigma-Aldrich, Czech Republic). Paraoxon (POX; *O,O*-diethyl-*O*-4-nitrophenylphosphate, 95% purity) was purchased from Dr. Ehrenstorfer (Augsburg, Germany). ^1^H- and ^13^C-NMR spectra were recorded at 300 and 75 MHz, respectively, on a Varian Mercury 300 spectrometer, using D_2_O as solvent. All experiments were carried out in compliance with the current law of Czech Republic.

### Synthesis

**Scheme 1:**
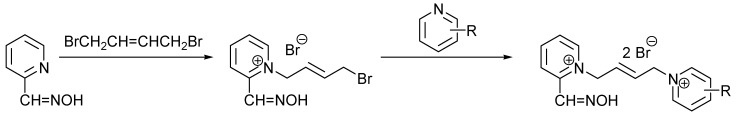
General scheme of the synthesis of novel acetylcholinesterase reactivators.

All newly prepared reactivators were prepared using the standard synthetic approach described previously by Musilek *et al*. [13,14]. As can be clearly seen from the reactivators´ structure, in the case of asymmetric reactivators there is the need to prepare them through the monoquaternary intermediate. To obtain the monoquaternary compound, there is a need of mild conditions to prevent a creation of symmetric bisquaternary compound (Scheme 1). NMR data together with melting points and yields of prepared compounds are listed below:

*trans-2,4′-Bis[(hydroxyimino)methyl]-1,1′-(but-2-ene-1,4-diyl)bispyridinium dibromide* (**K053**): yield 68%; m.p. 194-196 °C; ^1^H-NMR δ: 5.52 (d, 2H, *J* = 4.5 Hz, CH_2_), 5.89 (d, 2H, *J* = 8 Hz, CH_2_), 6.03 (dt, 1H, *J* = 16, 4.5 Hz, CH=), 6.34 (dt, 1H, *J* = 16, 8 Hz, CH=), 8.07 (t, 1H, *J* = 6 Hz, arom H-5), 8.21 (d, 1H, *J* = 6 Hz, arom H-3), 8.24 (d, 2H, *J* = 6 Hz, arom H-3′, H-5′), 8.38 (s, 1H, CH=N), 8.61 (t, 1H, *J* = 6 Hz, arom H-4), 8.63 (s, 1H, CH=N), 8.85 (d, 1H, *J* = 6 Hz, arom H-6), 8.87 (d, 2H, *J* = 6 Hz, arom H-2′, H-6′). The signals of =NOH disappeared in deuterated solvent; ^13^C-NMR δ: 48.37 (CH_2_), 62.23 (CH_2_), 126.94 (CH=), 126.96 (CH=), 127.02 (CH-3′,5′), 129.90 (CH-3), 132.85 (CH-5), 146.95 (CH-2′,6′), 146.99 (CH-6), 148.18 (C-4′), 148.21 (CH-4), 148.74 (CH=N), 151.41 (CH=N), 157.37 (C-2).

*trans-3,4-Dicarbamoyl-2′-(hydroxyimino)methyl-1,1′-(but-2-ene-1,4-diyl)bispyridinium dibromide* (**K054**): yield 55%; m.p. 209-211 °C; ^1^H-NMR δ: 5.40 (d, 2H, *J* = 7 Hz, CH_2_), 5.54 (d, 2H, *J* = 5 Hz, CH_2_), 6.04 (dt, 1H, *J* = 16, 7 Hz, CH=), 6.42 (dt, 1H, *J* = 16, 5 Hz, CH=), 8.09 (t, 1H, *J* = 6 Hz, arom H-5′), 8.34 (d, 1H, *J* = 6 Hz, arom H-3′), 8.43 (d, 1H, *J* = 7 Hz, arom H-5), 8.59 (t, 1H, *J* = 6 Hz, arom H-4′), 8.64 (s, 1H, CH=N), 8.86 (d, 1H, *J* = 6 Hz, arom H-6′), 9.11 (d, 1H, *J* = 7 Hz, arom H-6), 9.25 (s, 1H, arom H-2). The signals of CONH_2_ and =NOH disappeared in deuterated solvent; ^13^C-NMR δ: 48.48 (CH_2_), 61.25 (CH_2_), 127.51 (CH=), 129.08 (CH=), 129.30 (CH-5), 129.91 (CH-3′), 130.27 (CH-5′), 135.86 (C-3), 144.12 (CH-6), 146.76 (CH-6′), 148.37 (CH-4′), 148.76 (CH-2), 149.88 (CH=N), 152.04 (C-4), 157.32 (C-2′), 167.90 (CONH_2_), 169.49 (CONH_2_).

*trans-2,2′-Bis[(hydroxyimino)methyl]-1,1′-(but-2-ene-1,4-diyl)bispyridinium dibromide* (**K068**): yield 60%; m.p. 196-199 °C; ^1^H-NMR δ: 5.28 (d, 4H, *J* = 5 Hz, 2 x CH_2_), 6.06 (t, 2H, *J* = 5 Hz, 2 x CH=), 8.08 (dt, 2H, *J* = 8, 1.5 Hz, 2 x arom H-5), 8.38 (dd, 2H, *J* = 8, 1.5 Hz, 2 x arom H-3), 8.60 (dt, 2H, *J* = 8, 1.5 Hz, 2 x arom H-4), 8.78 (s, 2H, 2 x CH=N), 8.87 (dd, 2H, *J* = 8, 1.5 Hz, 2 x arom H-6). The signal of =NOH disappeared in deuterated solvent; ^13^C-NMR δ: 48.53 (CH_2_), 127.45 (CH=), 129.80 (CH-3), 130.53 (CH-5), 145.01 (CH-6), 148.57 (CH-4), 148.74 (CH=N), 157.31 (C-2).

*trans-4′-tert-Butyl-2-(hydroxyimino)methyl-1,1′-(but-2-ene-1,4-diyl)bispyridinium dibromide* (**K071**): yield 47%; m.p. **>** 300 °C; ^1^H-NMR δ: 1.25 (s, 9H, 3 x CH_3_), 5.22 (d, 2H, *J* = 5 Hz, CH_2_), 5.84 (d, 2H, *J* = 8 Hz, CH_2_), 6.04 (dm, 1H, *J* = 16 Hz, CH=), 6.32 (dm, 1H, *J* = 16 Hz, CH=), 8.02-9.25 (m, 8H, arom), 8.64 (s, 1H, CH=N). The signal of =NOH disappeared in deuterated solvent; ^13^C-NMR δ: 31.55 (CH_3_), 38.45 (C), 48.38 (CH_2_), 64.37 (CH_2_), 126.61 (CH-3′,5′), 127.47 (CH=), 127.54 (CH=), 128.30 (CH-3), 129.84 (CH-5), 142.96 (CH-2′,6′), 146.05 (CH-6), 146.69 (CH-4), 148.70 (CH=N), 157.94 (C-2), 166.88 (C-4′).

### Biochemical 

Purity of novel compounds was checked once again using TLC technique and HPLC technique immediately prior the experiment [15,16]. Reactivation activity of the synthesized reactivators was tested using our *in vitro* reactivation test [9]. A short description of this method is summarized here: human erythrocyte AChE or plasma BuChE were inhibited by solution of paraoxon to 5% of their original activity. Time of enzyme inhibition with paraoxon (2 hours, corresponding to 7 × T_1/2_) was calculated from experimentally determined half life (T_1/2_) of reaction between enzyme and paraoxon. Then, the inhibited enzyme was incubated for 10 min with a solution of reactivator at concentration 10^-4^ M and 10^-5^ M. Activity of AChE (BuChE) was measured spectrophotometrically by modified method according to Ellman with acetylthiocholine (butyrylthiocholine) as substrate [17]. The reactivation potency was calculated from the formula:

%R = (1 − (a_0_ − a_r_)/(a_0_ − a_i_)) × 100

where %R is percent of reactivation, a_0_ is activity of intact enzyme, a_i_ is activity of inhibited enzyme and a_r_ is activity of reactivated enzyme minus oximolysis and spontaneous hydrolysis. Each measurement was repeated three times and was conducted under standard laboratory temperature (25 °C). Calculations were performed using software GraphPad Prism version 4.00 for Windows, GraphPad Software, San Diego California USA (www.graphpad.com). 

## Figures and Tables

**Table 1:** Potency of the tested oximes to reactivate POX- inhibited human erythrocyte AChE and plasma BuChE at concentrations 100 µM and 10 µM. (%, mean value of three independent determinations, time of inhibition by paraoxon 120 min; time of reactivation by AChE reactivators - 10 min; pH 7.4; temperature 25 °C).

	Reactivation (%)							
	AChE				BuChE			
Concentration	100 µM		10 µM		100 µM		10 µM	
Reactivator	Mean	S.D.	Mean	S.D.	Mean	S.D.	Mean	S.D.
Pralidoxime^6^	18.0	0.7	1.3	0.7	5.5	0.1	1.0	0.2
Obidoxime^6^	96.9	0.7	59.4	0.7	9.9	0.3	2.2	0.3
HI-6 ^6^	16.1	0	3.9	0.7	2.3	0.2	0.8	0.4
K053	21.4	1.4	7.3	0.8	10.7	0.6	1.4	0
K054	0	0	0.4	0	0.2	0	0.2	0
K068	5.4	1.0	1.5	0.4	10.4	0	1.7	0
K071	0	0	0	0	0	0	0	0
